# Anatomical, functional, physiological and behavioural aspects of the development of mastication in early childhood

**DOI:** 10.1017/S0007114513002699

**Published:** 2013-09-24

**Authors:** Benjamin J. D. Le Révérend, Lisa R. Edelson, Chrystel Loret

**Affiliations:** Nestlé Research Center, Vers-Chez-les-Blancs, CH 1000-26, Lausanne, Switzerland

**Keywords:** Digestion, Oral processing of food, Mastication, Children, Eating habits

## Abstract

Mastication efficiency is defined as the efficiency of crushing food between the teeth and manipulating the resulting particles to form a swallowable food bolus. It is dependent on the orofacial anatomical features of the subject, the coordination of these anatomical features and the consistency of the food used during testing. Different measures have been used to indirectly quantify mastication efficiency as a function of children's age such as observations, food bolus characterisation, muscle activity measurement and jaw movement tracking. In the present review, we aim to describe the changes in the oral physiology (e.g. bone and muscle structure, teeth and soft tissues) of children and how these changes are associated with mastication abilities. We also review previous work on the effect of food consistency on children's mastication abilities and on their level of texture acceptance. The lack of reference foods and differences in testing methodologies across different studies do not allow us to draw conclusions about (1) the age at which mastication efficiency reaches maturity and (2) the effect of food consistency on the establishment of mature mastication efficiency. The effect of food consistency on the development of children's mastication efficiency has not been tested widely. However, both human and animal studies have reported the effect of food consistency on orofacial development, suggesting that a diet with harder textures enhances bone and muscle growth, which could indirectly lead to better mastication efficiency. Finally, it was also reported that (1) children are more likely to accept textures that they are able to manipulate and (2) early exposure to a range of textures facilitates the acceptance of foods of various textures later on. Recommending products well adapted to children's mastication during weaning could facilitate their acceptance of new textures and support the development of healthy eating habits.

The development of feeding skills is a complex process influenced by many factors^(^ ^1^ ^)^. Therefore, feeding skills have been investigated by two fields of science: (1) the behavioural science of feeding and (2) the biomechanics of feeding (e.g. chewing and swallowing), with particular emphasis on the first approach.

Foods intended to be fed to infants and toddlers are currently recommended based on motor and eating skills described by speech-language pathologists and expert feeding specialists^(^ ^2^ ^)^. Most of the recommendations are based on the observations of children during feeding. The biomechanical characterisation of mastication and its development has been less thoroughly addressed, even though it could bring new insights into child weaning and eating habits.

In the present review, we focus on the development of mastication between birth and age 6 years and its impact on mastication abilities (compared with mature adult mastication) and food acceptance. We selected this age range for two reasons: (1) at age 6 years, all deciduous teeth would have erupted and none would have been shed yet and (2) the WHO uses this age as the end of the first growth phase for height^(^ ^3^ ^)^. Although, strictly speaking, ‘to masticate’ is to grind and pulverise food inside the mouth, using the teeth and jaws^(^ ^4^ ^)^, this definition is extended here to the action of forming a swallowable food bolus, even if it is prepared by the mechanical action of the gums and tongue or the enzymatic action of saliva. We aim to describe the changes in the oral physiology (e.g. bone and muscle structure, teeth and soft tissues) of children and how these changes are associated with mastication abilities. Finally, we review previous work on the effect of food consistency on children's mastication abilities and on their level of texture acceptance.

Supporting the development of efficient and thorough mastication during weaning and early childhood could lead to many benefits in adulthood. There is a clear nutritional benefit in the development of efficient mastication. Thorough mastication is the trigger of many cephalic phase responses leading to endocrinal pathways influencing, for example, satiation processes that lead to a reduction in overeating^(^ ^5^ ^,^ ^6^ ^)^. In addition, the decrease in particle size in the bolus leads to nutritional advantages, which have been demonstrated *in vitro* ^(^ ^7^ ^)^ and *in vivo* ^(^ ^8^ ^,^ ^9^ ^)^, with smaller particle size allowing faster macronutrient hydrolysis and better molecular diffusion from the food to the lumen, both resulting in better nutrient uptake.

## Mastication apparatus and its development

The mastication apparatus is composed of four major components: bones, muscles, teeth and soft tissues, which are described below. It is important to first briefly describe how each of these components affects mastication. Mobile soft tissues such as the tongue, lips and cheeks ensure that the food is placed within the occlusal contact area, maximising chances of breakage. Muscle growth and coordination are needed to apply force on the bones and teeth so that fracturing is possible. Bone (maxilla and mandible) growth provides more space for the eruption of teeth, increasing tooth/food contact, and more space in the oral cavity to fracture larger food pieces as well as supports the increased force from stronger muscles. The eruption of teeth increases the amount of contact between the teeth and the food and results in teeth of different shapes so that force can be converted into different levels of stress (by modulating the topology of the tooth/food contact) to fracture different types of foods and achieve an appropriate final bolus particle size. The development of the mastication apparatus thus allows a wider variety of foods and textures to be processed by the mouth and thus improves the nutritional quality.

### Mastication apparatus

**Fig. 1:**
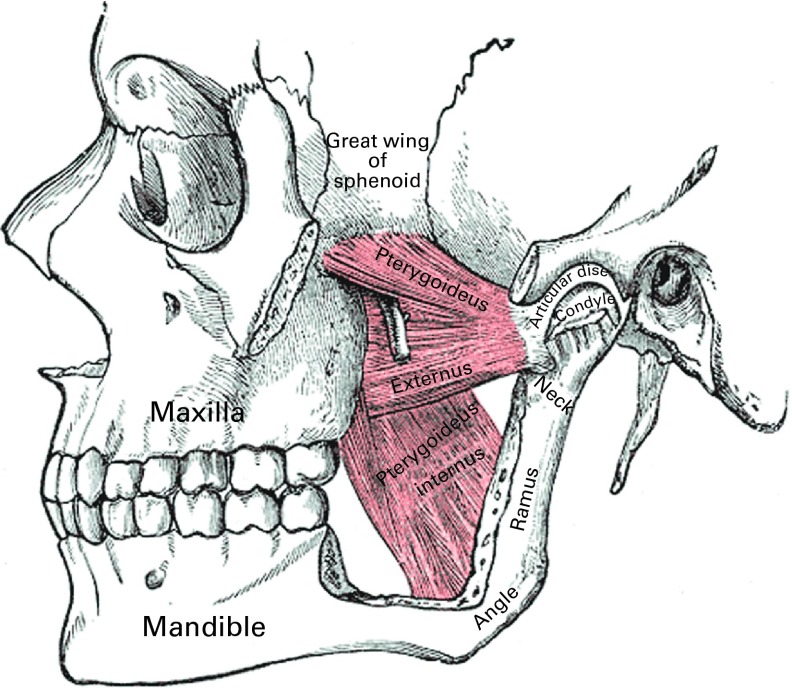
Bones and muscles involved during mastication^(^ ^10^ ^)^.

Bones involved in mastication are the maxilla (upper jaw) and mandible (lower jaw). The palate delimits the lower part of the maxilla. The gap between the palate and the mandible defines the oral cavity. The mandible and maxilla are joined together via the temporomandibular articulation (see Fig. 1).

Mastication movements are executed using muscles connected to the maxilla and mandible^(^ ^10^ ^)^:

(1) The temporalis, masseter and medial pterygoid are responsible for the occlusion of the mandible against the maxilla (elevators).
(2) Digastric, milohyoid and geniohyoid are responsible for the opening of the oral cavity (depressors).
(3) The lateral pterygoid assists in the opening of the mouth, but its main action is to draw forward the mandible so that the inferior incisors are projected in front of the upper ones; in this action, it is assisted by the medial pterygoid.
(4) The posterior fibres of the temporalis retract the mandible.
(5) If the lateral and medial pterygoids of one side act, the corresponding side of the mandible is drawn forward, leading to lateral movements. This action typically occurs during lateral chewing of foods.

The anchoring of these muscles in the craniofacial bone structure is also shown in Fig. 1. One can see in the figure that both the temporalis and masseter muscles are anchored on the maxillary and mandibular bones, allowing a rotational action at the temporomandibular joint during occlusion. The mandible and maxilla are the anchor points for the deciduous or primary teeth in children (*n* 20) and permanent teeth in adults (*n* 32). In children aged less than 36 months, dentition is composed of deciduous teeth only: incisors, canines and molars. These teeth serve different purposes: incisors are for cutting and canines are for cutting and tearing, while molars are mainly for chewing and shearing.

Finally, soft tissues in the oral cavity, such as the tongue, lips and cheeks, are also of importance in the manipulation of food during oral processing: maximising chewing efficiency by acting as moving boundaries ensuring bolus control in the oral cavity^(^ ^11^ ^)^. The tongue is a large bundle of striated muscles on the floor of the mouth.

### Development of the mastication apparatus with age

The mastication apparatus is not static over the course of a child's development. All of its major components (bones, muscles and teeth) are subject to a range of changes during the growth of infants and toddlers.

**Fig. 2:**
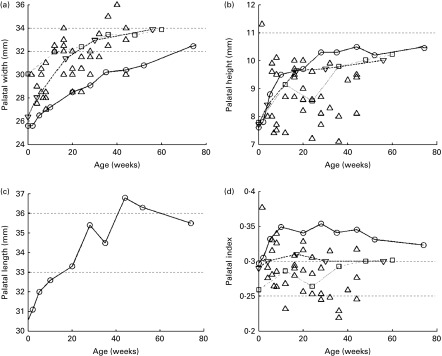
Palatal (a) width, (b) height (or depth), (c) length and (d) index evolution during the first few years of life. 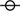, Bakwin; ◇, Denzer; 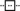, Hohoff; 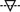, Procter.

If one considers the bone structure, the dimensions of the palatal arch seem to be an obvious measure of bone development. In most measurements of the palatal arch dimensions (width, height and length) that have been reported since the early decades of the twentieth century, very simple techniques (essentially a ruler or caliper) have been used^(^ ^12^ ^–^ ^14^ ^)^. Currently, more complex methods involving laser three-dimensional scanning of dental polymer casts are being used^(^ ^15^ ^)^ as well as magnetic resonance imaging^(^ ^16^ ^)^, although magnetic resonance imaging is not geared towards the imaging of bone tissue and thus could be less accurate than the previously mentioned techniques. Most of the data have been collected for children during their first year of life. In Fig. 2, the sets of data plotted against one another are shown.

For the palatal width (*P*_w_; Fig. 2(a)), all studies have reported average growth from 26 mm at birth to 32 mm at 12 months of age. Redman *et al.* ^(^ ^17^ ^)^ reported a width of 31 mm at the age of 6 years. The palatal height (*P*_h_; Fig. 2(b)) has been reported to increase from 6·5 to 11·5 mm in all the studies, with a mean value of about 9 mm. Hohoff *et al.* ^(^ ^15^ ^)^, Bakwin & Bakwin^(^ ^13^ ^)^ and Procter *et al.* ^(^ ^14^ ^)^ reported similar growth from 8 to 10 mm from birth to 12 months of age, and Denzer^(^ ^12^ ^)^ did not report such growth and data are scattered about 9 mm at all ages. Knowing the palatal width and height, the authors determined the palatal index (Fig. 2(d)), which is the ratio of the palatal height to the palatal width, giving a quantitative indication of the palatal shape.

Oddly, the palatal length (*P*_l_) is not as widely reported as the other two dimensions. Only Bakwin & Bakwin^(^ ^13^ ^)^ reported its growth over the first 12 months of life, increasing from 25·5 to 32·5 mm (Fig. 2(c)) and 43·6 mm at 6 years of age.

Against the maxilla (upper jaw) is the mandible (lower jaw). The mandible grows from 30 to 55 mm between birth and 6 years of age^(^ ^16^ ^)^. It is interesting to note that both maxilla and mandible grow in length by a similar amount of about 20–25 mm. When comparing the palatal and mandibular dimensions of infants^(^ ^12^ ^,^ ^13^ ^,^ ^15^ ^,^ ^16^ ^)^ with those of older children^(^ ^16^ ^,^ ^17^ ^)^ and adults^(^ ^16^ ^,^ ^18^ ^)^, it appears that the bone dimensions (palatal width, height and length and mandibular length) seem to evolve as much over the first year of life as they do between 1 year of age and puberty, after which growth continues until the bones reach the adult dimension by 18 years of age. This suggests that a high lever exists on orofacial anatomy modelling during the weaning period, hence the importance of offering appropriate textures at this age to support orofacial growth.

In addition to the development of the bone structure, the muscles acting on the jaw during occlusion also evolve during the first few years of child development. A common variable to measure this evolution is the thickness of the temporalis and masseter muscles using ultrasound imaging techniques. Masseter muscle thickness has been reported to be 9·47 (sd 0·95) mm at 59 months of age at rest, increasing up to 10·03 (sd 0·94) mm for children aged 73 months. This difference is significant at 1 %^(^ ^19^ ^)^. In contrast, no difference in masseter muscle thickness has been observed in the maximal intercuspal position, and no difference in temporalis muscle thickness (at rest and in maximal intercuspal position) has been observed as a function of age. No data are available for younger children. For adults, thicknesses reported are of the order of 13 mm for the masseter and 14 mm for the temporalis^(^ ^20^ ^)^; these differences in muscle thickness between children and adults should be correlated with a steady increase in bite force (see the Bite force section).

The number and type of teeth also change with children's age^(^ ^21^ ^)^: the central incisors erupt between 8 and 12 months of age; the lateral incisors erupt between 9 and 18 months; the first molars erupt between 13 and 19 months; the canines erupt between 16 and 22 months; the second molars erupt between 23 and 33 months. Changes in the number and state of the teeth influence children's jaw stabilisation and occlusion.

Regarding soft tissue development, the breadth of the mouth has been found to increase between 6 weeks and 36 months from 34·1 to 43·5 mm (closed mouth) or 28·5 to 36·9 mm (open mouth). In both cases, this represents about a 30 % increase, which also indicates that more room is available for food during development. Also the tongue and lips undergo a transformation from undifferentiated movements to more refined movements, which are necessary for bolus formation and propulsion^(^ ^22^ ^)^. In addition to improving the coordination of the motion of the tongue, the tongue muscles also increase in length from 6 to 9 cm between birth and 6 years of age^(^ ^16^ ^)^.

From the perspective of child weaning, such data are useful and should be investigated thoroughly. It would make it possible to determine the maximum food size to offer to a child of a certain age as well as to evaluate the available volume for food bolus formation and the force available to break the food down. This development of the physiological features of the children (i.e. bones, muscles and teeth) certainly influences their mastication abilities. The evolution of mastication abilities with age is described in the following section and links are made to the physiological oral characteristics of children as a function of age.

## Development of mastication efficiency with age

Mastication aims to decrease particle-size distribution in the food bolus and forms a cohesive bolus with the saliva in order to facilitate swallowing^(^ ^23^ ^–^ ^25^ ^)^. Thus, mastication efficiency can be defined as one's capacity to grind or pulverise food material to form a swallowable bolus^(^ ^26^ ^)^. Carlsson^(^ ^26^ ^)^ noted that there are several physiological factors that influence mastication efficiency, such as the state of dentition (number of teeth), occlusion contact area, bite force, and ability to control masticatory muscles for efficient contraction^(^ ^27^ ^)^ and soft tissues (tongue, lips and cheeks) to manipulate the bolus and place it in the occlusion area. This definition is widely used in the field, and we thus accept it as a point of reference. Different methods have been used to investigate children's mastication abilities, including (1) visual observation of the time of mastication and number of chews, (2) tongue, lip and (3) jaw movements, (4) muscle activity, (5) bite force and finally (6) characterisation of food bolus destructuration during food consumption.

### Visual observation of the time of mastication and number of chews

The first and most obvious method to measure chewing efficiency is to monitor the number of chewing cycles or time necessary for oral processing before swallowing and to determine the chewing frequency (times/cycle). This approach has been particularly popular for studying mastication in children as it is non-invasive and easy to implement. Using this method, Gisel^(^ ^28^ ^)^ reported an increase in mastication efficiency between 6 months and 2 years of age, depending on food texture. It was shown that for purées and soft solids (gelatine pieces), little improvement of chewing time and number of cycles occurred after 6 or 8 months of age, respectively. In contrast, for harder foods (Cheerios; General Mills), the chewing time decreased from 40 to 15 s and the number of chews before swallowing decreased similarly from thirty to fifteen between 6 months and 2 years of age. Earlier work by Gisel^(^ ^29^ ^)^ indicates that maturity for a specific texture has been achieved when the time taken to chew a bite of food remained constant across a given age range. Therefore, their data suggest that eating maturity was accomplished at 6 and 8 months of age, respectively, for the purée and the soft solids. However, for more solid textures, an increase in efficiency continued through the oldest participants aged 24 months, suggesting that maturity was not yet reached for this texture. This suggests that after 24 months of age, cereal-like textures still challenge the mastication abilities of children and thus support the development towards adult mastication. The authors also observed that the strategies used to chew the solid texture varied greatly among 6-month-olds. Some infants would let saliva soften it and then initiate swallowing through suckling motions. Others attempted to munch on it. From 8 months onwards, ‘munching’ was firmly established, meaning that food was crushed by raising and lowering the lower jaw, without a rotary component. Similar data were acquired for children between 2 and 8 years of age in a series of studies^(^ ^29^ ^–^ ^31^ ^)^, where it was found that both chewing time and number of chewing cycles decreased during the age range studied, which was interpreted as a continuous improvement with age. The time per chewing cycle frequency varied in the range 0·8–1·2 Hz depending on the food, but remained constant across age groups. In addition, it was found that the main difference in texture lay between solids (raisins and crackers) and liquid foods (applesauce). The chewing time was much shorter for the applesauce, and the frequency of chewing was lower (1·2 Hz for solids and 0·8 Hz for liquids) as well, showing that liquid foods may require more soft tissue manipulation between successive bites.

Gisel's team also investigated the effect of bite size on mastication abilities in typically developing children from 6 months to 2 years of age^(^ ^29^ ^,^ ^32^ ^)^. They hypothesised that the changes in facial structures of children during this period allow the ingestion of larger bites of food. However, they were not able to prove it. A significant effect of bite size between gelatine cubes of 10 × 4 × 4 mm and 5 × 4 × 4 mm was observed only for children of 8 and 18 months of age. At 6, 10, 12 and 24 months of age, no difference could be observed. They may have used products that were too small compared with the oral cavity volume (about 10 % of the palatal volume, which can be estimated from the above-reviewed literature), making them swallowable with minimal oral processing. Given the literature already reviewed in the present article, it would be relevant to more carefully consider the sizes of the food pieces used during such studies to yield more meaningful results. Feeding food pieces that are similar to the final size of particles in a food bolus is not likely to promote sufficient chewing action for the masticatory function to be assessed.

Such methods, although interesting from a developmental perspective, do not offer insight into how food is processed in the mouth, but aim to understand the degree of maturity of the masticatory function. However, given the importance of the textures used in the outcome of the tests, a series of models or simple food systems should be agreed upon and used by investigators so that comparisons between studies can be made.

### Tongue and lip motion

The movements of the tongue and lips undergo a transformation from synergistic, undifferentiated movements in infants to differentiated and refined movements required for biting, chewing, and bolus formation and propulsion in toddlers and young children.

The motor development of the lips has been reported to be associated with the overall development of feeding in a few studies. Stolovitz & Gisel^(^ ^33^ ^)^ investigated the circumoral movements (lips and cheeks) in responses to three different food textures (applesauce, gelatine dessert and Cheerios) in children aged 6 months to 2 years using visual observations during anticipation of food and removal of food from a spoon as well as the initiation of chewing and swallowing. Closing of the mouth to chew and lip occlusion about the spoon to remove food increase as children get older. This behaviour develops earlier for applesauce and gelatine desserts than for solid textures. Younger children prefer biting the spoon than using their lips, and this behaviour was explained by a higher need for trunk stabilisation during feeding at an early age. These observations were made by quantitative measurements of the closing pressure of the lips during feeding, using a strain gauge embedded in a spoon^(^ ^34^ ^)^. Lip pressure was found to increase steadily from 5 months to 3 years of age and to increase slightly from 3 to 5 years.

Furthermore, the initiation of chewing becomes more efficient as the tongue becomes more mobile and independent of the jaw, thus allowing control and manipulation of the food. Around 4–6 months of age, food is mashed by the tongue by an upward/downward motion^(^ ^35^ ^)^. Stolovitz & Gisel^(^ ^33^ ^)^ observed that at 10 months, children began to move solid textures from one side of the mouth using lateral movements of the tongue. Meyer^(^ ^36^ ^)^ described an elevation of the tongue tip for better bolus control. The sides of the tongue form a central groove, which becomes deeper with increasing age. Only observational data have been reported on tongue movements during eating due to technical constraints. Tongue movements could be observed using (1) videofluoroscopy, but for young healthy children, this method is considered too invasive due to the use of X-ray and a contrast agent, (2) articulography or electropalatography^(^ ^37^ ^,^ ^38^ ^)^; however, magnets need to be positioned on the tongue, and this method may be too uncomfortable for young children, and (3) ultrasound imaging, which has been used for children aged below 6 months to follow tongue movements during breast-feeding^(^ ^39^ ^)^ or for older children (6–12 years) with cerebral palsy^(^ ^40^ ^)^ during liquid consumption.

### Jaw motion

Tracking the kinematics of jaw movements is one non-invasive physiological measurement of chewing patterns^(^ ^41^ ^–^ ^45^ ^)^. Efforts devoted by Dr Moore, Dr Green and Dr Steeve in the last decade in this field have offered some new insights into the development of chewing during the first few years of life.

**Fig. 3:**
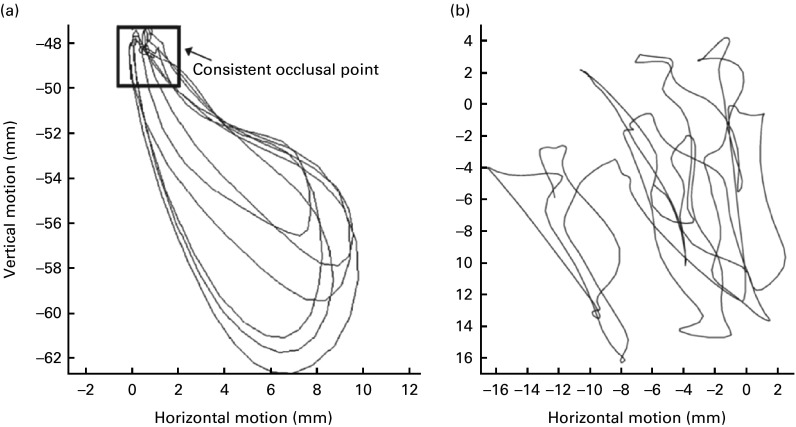
Excursion analysis using a (a) mature chewing sequence and (b) 12-month-old chewing sequence^(^ ^42^ ^)^.

Focusing on the description of chewing patterns, Wilson & Green^(^ ^42^ ^)^ followed a cohort of eleven children longitudinally from 9 to 30 months of age and measured the changes in their mastication kinematics during oral processing of two food consistencies: regular (e.g. Cheerios dry cereal) and purée (e.g. applesauce). In this study, the researchers reported that at 30 months of age, children still produced neither a rotary jaw movement nor a consistent occlusal point (the position where the jaw is fully closed). This is shown in Fig. 3. One can see what is typically expected from a mature chewer in Fig. 3(a) and the trace obtained for a 12-month-old child in Fig. 3(b). Parameterisation of these data (e.g. angle of the first component of the two-dimensional ellipse) showed no major improvement of the horizontal movement of the jaw during chewing until 24 months of age, and movements were not comparable with adult measurements at 30 months of age. This study is thus particularly interesting within the framework of the present review as it failed to support the dogmatic view, based primarily on video recording or direct observations of chin movements, that the rotary jaw movement exhibited by adults was acquired by 24 months of age. The literature describing chewing development suggests that at 4–6 months of age, jaw movements are simple elevations, assisted by actions of the lips and tongue. The next stage in the development of chewing is then marked by the emergence of lateral jaw motion to finally reach a rotary jaw movement, which is the sign of mature mastication at the age of 24–30 months^(^ ^11^ ^,^ ^24^ ^,^ ^35^ ^,^ ^36^ ^,^ ^46^ ^,^ ^47^ ^)^.

The type of food used in their study could also be one of the reasons for Wilson & Green^(^ ^42^ ^)^ not observing circular rotational movements. Indeed, Takada *et al.* ^(^ ^48^ ^)^ investigated the effects of food consistency on jaw movements during chewing in older children (average 11 years) and showed that lateral excursion of the jaw was only seen when chewing hard jelly. The Cheerios used as a hard reference food may not have been hard enough to stimulate a rotational movement. However, Wilson & Green^(^ ^42^ ^)^ showed that by the age of 18 months, there were significant differences across consistencies (*V*_Cheerios_>*V*_Purée_) in vertical velocity components. Although bite force was not explicitly measured, the authors suggested that children at 18 months of age might have learnt to regulate bite force. With the emergence of teeth, the sensory perception of texture may have refined and could explain this differentiation in mastication behaviour between the two food consistencies. The general decrease of jaw closing speed with age may also be a response to the acquisition of finer control of the lips and tongue, as well as the emergence of teeth. Indeed, it is well accepted that teeth, and particularly molars, provide a source of biomechanical stability to the jaw^(^ ^49^ ^)^. Another recent article by Wilson *et al.* ^(^ ^43^ ^)^ has looked at the introduction of foods in very young subjects (4–6 months of age, with an average exposure to purées for 2 weeks) compared with 7-, 12- and 35-month-olds and adults. The findings from this article suggest that there is a developmental timeline that starts at ages as low as 7 months. In addition, distinct differences in chewing measures (chewing duration, frequency and number of chews) between the 35-month-olds and the adult groups regardless of the consistency (purée, semi-solids and solids according to the National Dysphagia Diet) suggest that mastication is not yet mature at 35 months of age. This suggests that even at this age children are yet to fully master foods of different textures and that the development of mature mastication through an appropriate texture at this age is an opportunity to support healthy future eating habits.

Beyond this last reference, very little is known on the effect of bolus consistency on masticatory kinematics and how immature mandibular control is adapted to accommodate the progressive introduction of new food consistencies in young subjects. Developing this knowledge could help in the weaning phase of a child and guide design of food products with structural properties that encourage the physiological development to reach efficient mastication.

### Muscle activity

Another physiological measure of the actuators of the mastication movements is the recording of the activity of the muscles involved in mastication using surface electromyography. These measurements of oromotor activity have been extensively used for tracking speech development^(^ ^44^ ^,^ ^50^ ^–^ ^52^ ^)^ and, in recent years, for characterising chewing movements of young children eating soft or semi-solid foods such as cooked vegetables, fresh fruits (grapes, apricot, banana and apple), Cheerios, candies (jelly beans and gummy bears), crackers, potato chips or cookies^(^ ^53^ ^,^ ^54^ ^)^.

**Fig. 4:**
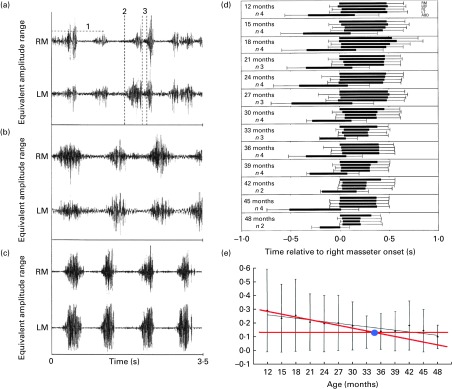
Electromyography traces of the left masseter (LM) and right masseter (RM) muscles from (a) 8-month-old, (b) 22-month-old and (c) adult^(^ ^54^ ^)^ subjects during a chewing sequence showing the increase in synchronicity between the agonist muscles. (d) A similar analysis^(^ ^53^ ^)^ can also be conducted with antagonist muscles. (e) The overlap time of activity between antagonist muscles is plotted (*y*= − 0·0043*x*+0·31). RT, right temporalis; LT, left temporalis; ABD, anterior belly of digastric.

Despite a wide variety of foods eaten, these studies showed that the development of adult-like chewing capabilities is characterised by a better synchronicity between the agonist muscles (temporalis and masseter) and between the antagonist muscles (temporalis/masseter and anterior belly of digastric) and a better defined onset and offset for bursts as well as a more constant amplitude during bursts with age (see Fig. 4). One can see in the figure that at 22 months of age (Fig. 4(b)), the electromyography traces are similar to the ones displayed during adult mastication (Fig. 4(c)), which led to the conclusion that at 22 months of age, children's muscle coordination may have reached maturity^(^ ^54^ ^)^. Fig. 4 also shows that the overlap in contraction of the antagonist muscles decreases with age, and a piecewise linear fit seems to show that this synchronicity between antagonists is mastered by 34 months of age^(^ ^55^ ^)^. This work also highlighted that the number of chewing cycles required to break down a food bolus decreases with age, as has been reported already by Gisel's work, although the chewing frequency does not evolve between 12 and 48 months of age. It should be noted that the (constant) frequency reported here was quite different (frequency varies in the range 1·5–2 Hz against 0·8–1·2 Hz for Gisel's work, see the previous section). This potentially highlights another limitation of visual observation to accurately monitor chewing activity.

To our knowledge, no study has been carried out on the effect of food consistencies on muscle activities during the development of chewing. Takada *et al.* ^(^ ^48^ ^)^ showed higher peak activities of the temporalis muscles during the consumption of hard jelly than during that of soft jelly but for older children (average 11 years).

### Bite force

**Fig. 5:**
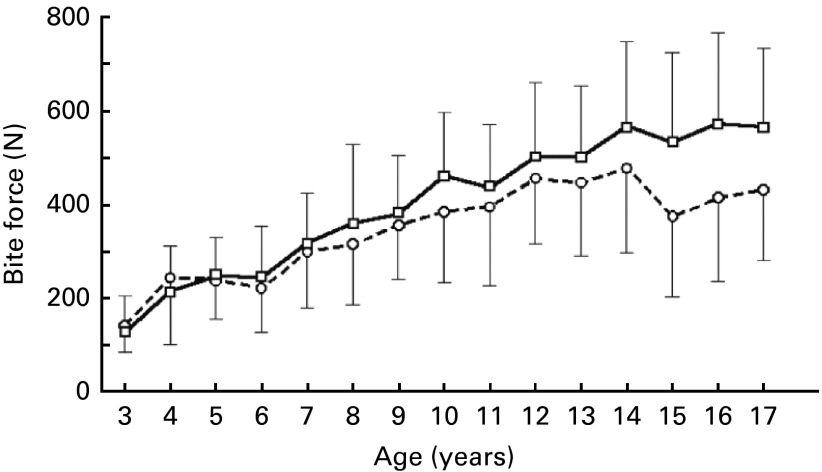
Bite force for male (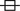) and female (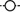) children^(^ ^61^ ^)^. Values are means, with standard deviations represented by vertical bars.

A direct translation of muscular activity is bite force, which could also be used to measure mastication abilities, as the capacity to break a piece of food depends on the force applied at its spatial boundaries. Bite force is typically measured using a force transducer, which can vary in technology being either electronic^(^ ^19^ ^,^ ^56^ ^–^ ^62^ ^)^ or analogue, using a spring-based strain gauge^(^ ^63^ ^)^. No bite force measurements are available for children under the age of 3 years. This is probably due to the fact that it is difficult to obtain such data in a reproducible manner as intra-individual variability may already be too high to find a statistically significant difference, and it is also impossible to instruct infants to bite as hard as possible on a force transducer. The single best reference in this respect is probably the database compiled by Kamegai *et al.* ^(^ ^61^ ^)^ reporting data from 2594 Japanese children aged between 3 and 17 years, measured in the molar region as shown in Fig. 5. These data indicate that the upper boundary for the age range that we are interested in is of the order of 220 N and plateaus between 4 and 6 years of age, supporting our choice of age range. It is interesting to note that none of the studies has reported a significant difference between male and female children, who only differ in bite force from puberty.

In addition to the evolution of the maximal bite force with age, one should consider the distribution of bite force across the dental arch, as depending on where the foods will be positioned in the mouth and the number of teeth available, the maximal bite force that could be applied by the children will differ. In all the studies mentioned in the present review^(^ ^23^ ^,^ ^26^ ^,^ ^63^, the bite force at the molars has been reported to be greater than that at the canines, which in turn is greater than that at the incisors. However, a few studies have reported the bite force distribution across the palatal arch for children^(^ ^64^ ^)^, for whom the molar bite force also seems to be higher than the incisor bite force.

Knowing the maximal force that a young child could apply at different positions in the dental arch would give guidance on food textures to be designed for children to promote the use of the full range of bite forces while maintaining safety regarding choking hazards.

### Food bolus destructuration during food consumption

Chew-and-spit experiments have been described by many authors such as Lillford^(^ ^65^ ^,^ ^66^ ^)^ and Lucas and co-workers^(^ ^67^ ^–^ ^69^ ^)^. As expected, most of this work has been done on young adults. For obvious reasons, collecting boli from infants seems to be impractical, as they cannot be instructed on how to complete the task.

Some researchers, however, reported the use of this technique for children aged between 3 and 5 years^(^ ^58^ ^,^ ^70^ ^)^, using Optosil (a typical dental impression gel that is commercially available) as the chewed material and image analysis to measure particle size. They reported a mean particle size of *D*= 4·6 mm. In another study^(^ ^60^ ^)^, 6- to 8-year-old children underwent a similar chewing test (Optosil, twenty chewing cycles) and similar values (*D*= 4 mm) were reported. In the same study^(^ ^60^ ^)^, bolus resulting from adult mastication was also reported to contain particles with a mean diameter of *D*= 2–3 mm. This would tend to show that there is an increase in mastication efficiency after 8 years of age, which was the oldest age group in Gisel's work, if not in time and in particle-size reduction abilities. Readers should note that results obtained from this technique seem to be highly variable, as a study carried out under the same condition has reported a mean diameter of *D*= 5·08 mm^(^ ^71^ ^)^ for particles in Optosil boli after twenty chewing strokes. Such data show that mastication efficiency does not seem to improve after 3 years of age, at least for softer solids. These data are thus in line with those already cited, as they tend to show that the oromotor skills are still improving after 6 years of age. The factors that are used to explain this increase in masticatory performance with age are mainly the mandibular size and bite force, which have been shown to increase with age. In our view, it seems that better particle-size reduction, if really improved, could also be attributed to better tongue skills, which will improve the positioning of the bolus in the occlusal area, as well as the emergence of the first molars increasing the number of teeth in contact and increasing the efficiency of the grinding process^(^ ^68^ ^,^ ^69^ ^)^. This would not have been necessarily measured by visual observations, as tongue movements are difficult to quantify this way (readers can refer to the relevant previous sections).

Due to the wide variety of methods that have been employed, the findings on changes in mastication abilities as a function of age and food served have been inconsistent, making it difficult to conclude on the age at which mastication is fully mature. A systematic approach with foods with well-known physical properties and the use of complementary measurement methods such as bite force measurement, video recording, jaw movement measurement, and muscle activity measurement would provide more conclusive data.

## Effect of food consistency on children's development (physiology and behaviour)

In the previous section, we summarised that the development of the mastication apparatus has an impact on oral food processing. It is also of interest to determine whether oral processing of different food consistencies has an impact on children's development. This includes, for example, orofacial growth (physiological) and texture acceptance (behavioural).

### Effect of food consistency on orofacial development

In this area, human studies, although interesting, lack the strength of evidence due to the ethical problems posed by an interventional study; however, animal literature is quite convincing due to more controlled conditions offered by interventional studies.

Animal studies have shown that a diet of liquidised or puréed food reduced the size of the masseter and temporal muscles as well as of the salivary glands of rats^(^ ^72^ ^)^ and had an impact on the motor performance of the jaw and tongue muscles^(^ ^73^ ^)^. A study on minipigs has also observed that the pigs fed a harder diet had larger temporalis and masseter muscles after 8 months of weaning and a better dentition as well as a higher and broader facial bone structure^(^ ^55^ ^)^. The authors suggested the implication of the weaning diet consistency for human orofacial development: ‘[…] it is apparent that the deliberate consumption of food items requiring vigorous chewing would constitute both responsible parenting and intervention. While it may be possible to develop and test clinically a product line that would enhance normal masticatory function, the practicality of this idea was considered and then rejected by Klatsky and Fisher [1953] long ago.’ In the twenty-first century, this approach seems to be more conceivable, as the needs for orthodontic treatment have risen in the last few decades^(^ ^74^ ^)^; this approach could be revisited at least from a health economics perspective.

There is quite a lot of literature exploring the relationship between muscle thickness or maximal bite force and craniofacial morphology^(^ ^74^ ^)^. It has been reported that subjects with thin masseters have a proportionally longer face, which could be due to the lack of both bone and muscle volume^(^ ^75^ ^)^ and lower bizygomatic and intergonial width^(^ ^56^ ^)^, while subjects with a higher bite force have a short lower anterior face height^(^ ^59^ ^,^ ^62^ ^)^, a small mid-face inclination, a large mandible and occlusal plane inclination^(^ ^17^ ^,^ ^57^ ^)^, and a smaller jaw, leaving less space for the eruption of teeth^(^ ^76^ ^)^. Hall^(^ ^77^ ^)^ also reported that for the face of a newborn to have a normal morphological appearance, contraction of the muscles involved in mastication and facial expression must occur to stimulate forward bone growth, cartilage growth and facial muscle bulk. It is hypothesised that the development of masticatory muscles could depend on the consistency of children's early diet^(^ ^78^ ^)^. Qualitative studies have been conducted to search for evidence of differences in orofacial development due to diet consistency. Studies based on different diets due to cultural differences, secular changes or living styles (non-urbanised people *v.* urbanised people) rather than on design seem to confirm the hypothesis that diet consistency can influence the orofacial growth. Larsson^(^ ^79^ ^)^ studied two populations of children, Swedish and Norwegian, and reported huge differences in feeding practices. Norwegian children were mostly breast-fed, started eating porridge at 4–5 months of age and, starting at 6 months of age, were fed dark, hard chewy bread to gnaw on. In contrast, the Swedish children were breast-fed for a shorter time period and mainly given food with little chewing resistance during the first 1 or 2 years of life. The authors reported that occurrences of posterior crossbite and narrow upper jaws were more common in the Swedish population than in the Norwegian children and explained these differences as being due to the different diets as well as to differences in pacifier/finger-sucking habits. Although this study showed a clear impact of food consistency on oral development, it also reported that Norwegian children neither needed a pacifier nor sucked their fingers, whereas some Swedish children did develop a pacifier or finger-sucking habit. The conclusion drawn on the effect of food consistency on oral development would have been more powerful without differences in terms of finger sucking, as this, of course, also influences oral development. Still, these results suggest an association between food consistency and oral development. Little *et al.* ^(^ ^80^ ^)^ explained the secular changes in craniofacial dimensions (narrower face, shorter face and smaller mandible in spite of an increase in overall body dimensions) among indigenous children in an isolated community in Mexico over an interval of 32 years (1968–2000) by a decrease in food (maize) coarseness or grit content. Corrucini & Choudhury^(^ ^81^ ^)^ reported significant differences in variability and in the prevalence of abnormality of several dental occlusal features among rural and urban male Bengali youths. Among a variety of socio-environmental factors determined through interviews, the masticatory resistance provided by unprocessed food exhibits the strongest independent contribution to the differences.

This idea is also supported by members of the orthodontic community^(^ ^82^ ^)^, who suggest that modern, softer foods are partly responsible for the functional atrophies of masticatory muscles and bone growth^(^ ^83^ ^,^ ^84^ ^)^.

### Effect of food consistency on texture acceptance

It has been described that the evolution of children's mastication apparatus has an impact on their willingness to accept textured foods^(^ ^85^ ^)^. Logically, at birth, infants can only process foods that only require swallowing: liquids. As their oromotor skills develop, they are able to process and thus accept soft solids by about 6 months of age and solids by about 10 months with the emergence of their first teeth. Chewy foods that require further breakdown only begin to be accepted by the age of 2 years, with the emergence of molars as well as the beginning of lateral chewing. As the mastication apparatus develops, an interest in exploring new sensory experiences, such as taste and texture, emerges.

It seems that children are more willing to accept foods that they can break down and chew easily. In two studies, it has been found that infants prefer puréed textures to lumpy or diced ones^(^ ^86^ ^,^ ^87^ ^)^, as these textures are easier to process. However, as children's mastication system matures, they become more interested in more complex textures, with toddlers preferring lumpy and diced textures to puréed ones^(^ ^87^ ^)^ and 12-month-olds with more teeth consuming more chopped carrots compared with their toothless peers^(^ ^86^ ^)^. Appropriate texture introduction through the course of weaning is also favourable for the development of texture acceptance. It is indeed reported that exposure early on to solid foods (before 10 months of age) reduced children's pickiness^(^ ^88^ ^)^ at 15 months of age and up to 7 years of age^(^ ^89^ ^)^. Similar findings have also been observed regarding exposure to a variety of tastes and flavours^(^ ^90^ ^,^ ^91^ ^)^. This could be associated with the idea summarised in the previous section, as early exposure to textures may boost early muscle development and thus make textured foods easier to process at a later stage, inducing preference.

As children's feeding behaviour matures, they show increased mobility of the tongue and improved jaw movement and can manipulate complex textures more easily. The relationship between the development of the mastication apparatus and the acceptance of food was also emphasised by a study on the preference of foods depending on the bite force of children aged between 7 and 12 years^(^ ^92^ ^)^. It was found that children who exhibited a higher bite force had a more positive attitude towards harder foods such as cabbage and celery compared with children with a lower bite force. The authors concluded, similarly to Ciochon *et al.* ^(^ ^55^ ^)^, that ‘[…] it is important to evaluate children's diet in relation not only to the nutritional and carcinogenicity aspects, but also in relation to its consistency, which may determine if a good biting ability will be acquired and subsequently influence the development of the masticatory system. To obtain larger bite force and occlusal contact area among elementary school children, […] awareness and appreciation of hard food should be promoted.’

Experiences with different textures early in life might facilitate infants’ acceptance of more complex textures at a later stage. Therefore, offering textures that are well adapted to the ability of children may improve their dietary choices in the future.

## Discussion and conclusions

In the present review, we have gathered insights into how the different physiological features of mastication evolve during the first few years of child development and how this can be linked to the establishment of mature mastication efficiency. Muscle and bone growth, dentition state, and lip and tongue development play a role, but each component matures at different rates.

Different authors have used different measures to indirectly quantify mastication efficiency as a function of children's age, such as observations, food bolus characterisation (particle-size distribution), muscle activity measurement (bite force and electromyography) and jaw movement tracking. These different studies have led to inconsistent conclusions on the age at which stable, mature mastication efficiency is reached, with estimates ranging from 8 months to 18 years. Furthermore, different products were used to record these data, making it difficult to compare results across studies.

Even when products used during testing were similar, conclusions about the age at which mature mastication efficiency is achieved varied depending on the method used. For purées and soft solids (gelatine pieces), observational chewing cycle studies identified mature mastication efficiency by 8 months of age and for harder solids (Cheerios) not earlier than 24 months of age. In contrast, studies using kinematics of jaw movements showed that before 18 months of age, a child could not adapt his or her jaw movements in response to similar consistencies. From 18 months onwards, jaw movements were more controlled. In addition, it is interesting to highlight that these two measurement techniques recorded vastly different ranges of chewing frequencies (0·8–1·2 and 1·5–2 Hz, respectively).

The lack of reference foods and differences in testing methodologies across different studies do not allow us to draw conclusions as to which method is best to characterise mastication efficiency or as to which foods are mastered to be processed at which age. This highlights the need for a complete experimental design including children of different age groups, well-controlled food sample sets and coupled testing methodologies. Results from such a study would provide valuable guidance for establishing public health policies and advice on the introduction of textures in early childhood.

The effect of food consistency on children's development of mastication efficiency has not been explored widely, and there would be potential benefits in investigating this area further, including by looking at consequences on orofacial development and eating habits. Both human and animal studies have reported the effect of food consistency on orofacial development, suggesting that a diet with harder textures enhances bone and muscle growth, which could indirectly lead to better mastication efficiency and potentially reduce the need for orthodontic treatment. This indicates that a range of carefully chosen foods could be used to promote the development of mastication capabilities.

Finally, it is also reported that (1) children are more likely to accept textures that they can manipulate easily and (2) an early exposure to a range of textures facilitates the acceptance of other textures later on. Offering products that are well adapted to a child's mastication ability during weaning could facilitate his or her acceptance of new textures and help the development of healthy eating habits.
